# Donor-Acceptor Copolymers with 9-(2-Ethylhexyl)carbazole or Dibenzothiophene-5,5-dioxide Donor Units and 5,6-Difluorobenzo[*c*][1,2,5]thiadiazole Acceptor Units for Photonics

**DOI:** 10.3390/nano13222939

**Published:** 2023-11-13

**Authors:** Věra Cimrová, Petra Babičová, Mariem Guesmi, Drahomír Výprachtický

**Affiliations:** Institute of Macromolecular Chemistry, Czech Academy of Sciences, Heyrovského nám. 2, 162 00 Prague 6, Czech Republicvyprachticky@imc.cas.cz (D.V.)

**Keywords:** donor-acceptor copolymers, 9-(2-ethylhexyl)carbazole, dibenzothiophene-5,5-dioxide, 5,6-difluorobenzo[*c*][1,2,5]thiadiazole, photophysics, electrochemistry, electroluminescence

## Abstract

Semiconducting polymers, particularly of the third generation, including donor-acceptor (D-A) copolymers, are extensively studied due to their huge potential for photonic and electronic applications. Here, we report on two new D-A copolymers, CP1 and CP2, composed of different electron-donor (D) units: 9-(2-ethylhexyl)carbazole or dibenzothiophene-5,5-dioxide, respectively, and of 4,7-bis(4′-(2-octyldodecyl)thiophen-2′-yl)-5,6-difluorobenzo[*c*][1,2,5]thiadiazole building block with central 5,6-difluorobenzo[*c*][1,2,5]thiadiazole electron-acceptor (A) units, which were synthesized by Suzuki coupling in the high-boiling solvent xylene and characterized. The copolymers exhibited very good thermal and oxidation stability. A copolymer CP1 with different molecular weights was prepared in order to facilitate a comparison of CP1 with CP2 of comparable molecular weight and to reveal the relationship between molecular weight and properties. The photophysical, electrochemical, and electroluminescence properties were examined. Intense red photoluminescence (PL) with higher PL efficiencies for CP1 than for CP2 was observed in both solutions and films. Red shifts in the PL thin film spectra compared with the PL solution spectra indicated aggregate formation in the solid state. X-ray diffraction measurements revealed differences in the arrangement of molecules in thin films depending on the molecular weight of the copolymers. Light-emitting devices with efficient red emission and low onset voltages were prepared and characterized.

## 1. Introduction

Semiconducting polymers, including donor-acceptor (D-A) copolymers, are extensively studied due to their potential for photonic and electronic applications, such as in light-emitting devices (LEDs), photovoltaic devices (PVDs), field-effect transistors, sensors, electrochromic devices, etc. [1,2,3,4,5,6,7,8,9,10,11,12,13,14,15,16,17,18,19,20,21]. Some of them could be used not only as active layers but also as transporting layers in organic or perovskite devices [22,23,24]. Recently, much attention has been focused on their use in organic solar cells. Power conversion efficiencies of up to 20% have been achieved in single-junction organic solar cells with bulk heterojunction active layers from blends of wide-bandgap polymer donors and non-fullerene small-molecule organic electron acceptors with a narrow bandgap [25,26,27,28] and higher efficiencies than 20% in tandem organic solar cells [29,30]. 

The D-A copolymers are prepared by the copolymerization of electron-donor (D) and electron-acceptor (A) monomers. The choice of the D and A units is important for the properties of D-A copolymers because they are influenced by the character of these units. The fine bandgap tuning is enabled due to the intrachain charge transfer (ICT) from the D to the A. Various D and A building blocks have been used for the syntheses of D-A copolymers. Thienothiadiazole- and benzothiadiazole-based units with the thiadiazole nucleus are among the most interesting A blocks [31,32,33,34,35,36,37,38,39]. The benzothidiazole and its derivatives are of interest, namely for the syntheses of D-A copolymers for photovoltaic applications [40,41,42,43,44]. On the other hand, carbazole derivatives belong to the interesting D blocks. In this work, we have focused on the syntheses and study of two new D-A copolymers possessing the interesting fluorinated benzothiadiazole as an A unit, which is in the center of the 4,7-bis(4′-(2-octyldodecyl)thiophen-2′-yl)-5,6-difluorobenzo[*c*][1,2,5]thiadiazole (ODTffBT) building block, and two different D units: 9-(2-ethylhexyl)carbazole (CzEH) and dibenzothiophene-5,5-dioxide (DBSO). Conjugated polymers with carbazole derivatives such as CzEH incorporated in the backbone exhibit interesting photophysical, electroluminescent, and electrochromic properties for photonic applications [45,46,47,48,49]. The CzEH carbazole-based D unit has stronger electron-donating ability compared with DBSO, which has been reported mainly as an A unit in a series of conjugated copolymers based on fluorene or carbazole [50,51,52,53,54,55,56,57]. The electron-withdrawing SO_2_ group is interesting because it reduces the electron density of the backbone and improves resistance against oxidation. The introduction of this unit can also effectively improve electron injection or collection in LEDs or PVDs. Further, copolymers containing heteroatoms are also of interest in combination with perovskites as additives or additional layers for their improved crystallization morphology, smoothness, and void-reducing coverage due to the enhanced interaction between grains in perovskite film that enhances device stability and performance [58,59,60,61,62,63,64,65,66]. The interaction between grains is enhanced by the possible coordination interaction of empty lead or tin orbitals with molecules containing heteroatoms such as oxygen or nitrogen, and also because the hydrogen atom in methylammonium or formamidinium can form hydrogen bonds with atoms of small radius and high electronegativity, such as fluorine [58,59]. 

**Figure 1:**
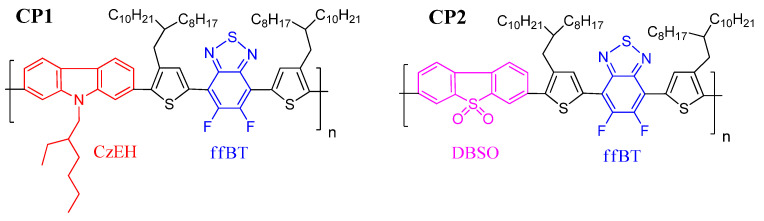
Chemical structure of the donor-acceptor (D-A) copolymers CP1 and CP2. The electron-donor (D) 9-(2-ethylhexyl)carbazole (CzEH) and dibenzothiophene-5,5-dioxide (DBSO) units are shown in red and magenta, respectively, and the electron-acceptor (A) 5,6-difluorobenzo[*c*][1,2,5]thiadiazole (ffBT) unit is shown in blue.

In this paper, we report on the synthesis and properties of the new copolymers, poly{4,7-bis(4′-(2-octyldodecyl)thiophen-2′-yl)-5,6-difluorobenzo[*c*][1,2,5]thiadiazole-5′,5′-diyl-*alt*-9-(2-ethylhexyl)carbazole-2,7-diyl} (CP1) and poly{4,7-bis(4′-(2-octyldodecyl)thiophen-2′-yl)-5,6-difluorobenzo[*c*][1,2,5]thiadiazole-5′,5′-diyl-*alt*-dibenzothiophene-5,5-dioxide-3,7-diyl} (CP2). Their chemical structure is displayed in Figure 1.

The copolymers consist of an ODTffBT block with the central 5,6-difluorobenzo[*c*][1,2,5]thiadiazole (ffBT) A unit and D units, either CzEH in CP1 or DBSO in CP2, which differ significantly in their electron-donating ability as mentioned above. Three CP1 copolymers with different molecular weights (CP1-a, CP1-b, and CP1-c) were prepared in order to obtain a suitable comparison of the properties of CP1 and CP2 copolymers as well as to determine the relationship between molecular weight and properties. The thermal stability, photophysical (absorption and photoluminescence (PL)), electrochemical properties, and electroluminescence (EL) were investigated with the aim outlined above of obtaining information about the influence of the structure and molecular weight on their properties. In addition, X-ray diffraction (XRD) measurements of thin films were performed to identify differences in the molecular arrangement of thin films, which are discussed in relation to the results of the photophysical and EL studies. The copolymers were tested as active layers in LEDs with regular and inverted structures, which were both characterized. They showed efficient red emission and low onset voltages. To the best of our knowledge, the CP1 and CP2 copolymers, including the study of their properties and LEDs, have not been reported before. Based on the obtained results, they show considerable potential for photovoltaic applications, especially in organic or perovskite solar cells. Their use in such applications is outlined.

## 2. Materials and Methods

### 2.1. Materials

Solvents and common chemicals were purchased from TCI (TCI Europe, N.V., Zwijndrecht, Belgium), VWR (VWR International s.r.o., Stříbrná Skalice, Czech Republic), and Lach-Ner (Lach-Ner, Ltd., Neratovice, Czech Republic). 4,7-Bis(5′-bromo-4′-(2-octyldodecyl)thiophen-2′-yl)-5,6-difluorobenzo[*c*][1,2,5]thiadiazole monomer (**3**), poly[3,4-(ethylenedioxy)thiophene]/poly(styrenesulfonate) (PEDOT:PSS) and [6,6]phenyl-C_71_-butyric acid methyl ester (PC_71_BM) were obtained from Ossila Ltd. (Ossila European Fulfillment Ltd., Belfast, Northern Ireland). 2,7-Dibromocarbazole, ethanolamine, zinc acetate dihydrate (99.999% trace metal basis), and 2-methoxyethanol (99.8% anhydrous) were purchased from Merck (Merck spol. s.r.o., Praha, Czech Republic). Tetrahydrofuran (THF) was refluxed (7 h) with LiAlH_4_ and distilled. 9-(2-Ethylhexyl)-2,7-dibromocarbazole and 9-(2-ethylhexyl)-2,7-bis(4,4,5,5-tetramethyl-1,3,2-dioxaborolan-2-yl)carbazole (**1**) were synthesized according to our previous papers [67,68,69]. 3,7-Bis(4,4,5,5-tetramethyl-1,3,2-dioxaborolane-2-yl)dibenzothiophene-5,5-dioxide (**2**) was synthesized analogously to procedures described in the literature [70,71]. A precursor zinc oxide (ZnO) solution was prepared by dissolving zinc acetate dihydrate (1 g) in 2-methoxyethanol (10 mL) and by adding a small amount of ethanolamine (0.28 mL) as a surfactant under vigorous stirring for 12 h under air for the hydrolysis reaction.

### 2.2. Sample Preparation

Copolymer thin films were prepared from toluene solutions for optical measurements by spin-coating onto fused silica substrates and electrochemical measurements by dipping on Pt wires. Films of the blends with PC_71_BM (1:3 wt.) were spin-coated from 1,2-dichlorobenzene (DCB) solutions. The film preparation was performed in a glove box under a nitrogen atmosphere (M. Braun Inertgas-Systeme GmbH, Garsching, Germany). The films were dried in a vacuum oven built into the glove box at 10^−3^ Pa and 373 K for 1 h. Two types of LEDs were prepared: 1. LEDs of regular structure (regular LEDs) having indium-tin oxide (ITO)/PEDOT:PSS hole- and calcium (Ca)/aluminium (Al) electron-transporting electrodes and 2. LEDs of inverted structure (inverted LEDs) having ITO/ZnO electron- and molybdenum oxide (MoO_3_)/silver (Ag) hole-transporting electrodes. The PEDOT:PSS layers (ca. 40 nm) were spin-coated onto ITO substrates under air and dried at 398 K for 15 min. The ZnO layers (ca. 30 nm) were prepared by spin-coating the precursor ZnO solution onto the ITO substrate and drying at 473 K under air. Then the prepared substrates were transferred into the glove box, and subsequently, the copolymer films were spin-coated and dried as described above. Regular LEDs were finished by vacuum–evaporation of 20 nm thick Ca on the top of the polymer layer and subsequently of 100 nm thick Al using an evaporator built into the glovebox. Inverted LEDs were finalized by vacuum evaporation of 10 nm thick MoO_3_ on the top of the polymer layer and, subsequently, of 100 nm thick Ag. The typical active area of the LEDs was 4 mm^2^.

### 2.3. Methods

An upgraded Bruker Avance DPX-300 spectrometer operating at 300.13 MHz and 75.45 MHz was used for the measurements of ^1^H and ^13^C NMR spectra. An internal standard was hexamethyldisiloxane. Fourier transform infrared (FTIR) spectroscopic measurements were performed using a Nicolet 6700 spectrometer (Thermo Scientific, Madison, WI, USA). Size exclusion chromatography (SEC) was used for the determination of weight- and number-average molecular weights (*M*_w_ and *M*_n_). THF was the mobile phase, and polystyrene standards were used for calibration. Evaporative light scattering detector PL-ELS-1000 (Polymer Laboratories, Ltd., Church Stretton, UK), pump Deltachrom (Watrex s.r.o., Prague, Czech Republic), autosampler Midas, and two columns PL gel MIXED-C LS, particle size 5 μm, were used in the experimental setup. The Perkin-Elmer TGA 7 Thermogravimetric Analyzer (PerkinElmer Instruments, Shelton, CT, USA) operated with Pyris 1 software version 10.1.0.0412 was used for thermogravimetric analysis (TGA) performed in nitrogen as well as in air at a flow of 50 mL min^–1^ and at a heating rate of 10 K min^–1^.

A Perkin-Elmer Lambda 35 UV-vis spectrometer (PerkinElmer Instruments, Shelton, CT, USA) was used for measurements of UV-vis spectra. PL spectra were measured using a Perkin-Elmer LS55 and FS5 fluorescence spectrophotometer (Edinburgh Instruments Ltd., Livingston, UK) equipped with an SC-30 integrating sphere module for quantum yield determination. A Spectra Pro 300i monochromator/spectrograph (Acton Research Corporation, Acton, MA, USA) with single photon-counting detection (SPEX, RCA C31034 photomultiplier) was used for measurements of EL spectra. Current-voltage characteristics of LEDs were measured using a Keithley 237 source measure unit (Keithley Instruments, Inc., Cleveland, OH, USA). EL intensity and voltage characteristics were recorded simultaneously. A silicon photodiode with integrated amplifier HUV-4000B (EG&G, Montgomeryville, PA, USA) connected to an Agilent 34401A multimeter (Agilent Technologies, Inc., Santa Clara, CA, USA) or luminance meter Minolta LS110 (Minolta Camera Co., Ltd., Osaka, Japan) was used for the light output detection. LED characteristics were measured under a nitrogen atmosphere. Layer thicknesses, which were in the range of 90–200 nm, were determined using a KLA-Tencor P-10 or P-17 profilometer (KLA-Tencor Corporation, Milpitas, CA, USA).

X-ray diffraction (XRD) experiments were carried out by means of an Explorer X-ray diffractometer (GNR Analytical Instruments, Agrate Conturbia, Italy). The measurements were performed in theta-2theta geometry in the range from 1.4 to 45 deg with an angle step of 0.01 deg and a counting time of 20 s in each step. The sample plane was horizontal and fixed. The X-ray source and the detector were placed on their own on a moveable arm with a radius of 600 mm. The copper X-ray tube was supplied at 30 mA at 40 kV. Soller slits were used to collimate the beam and a Ni filter to monochromatize it. The dominant wavelength was Cu Kα (1.54 Å). Diffraction was detected by a Mythen 1 k Strip detector (CeleriX, Baden-Daettwil, Switzerland) with an active area of 62 × 8 mm and a 1280 tailorable number of pixels at a sample−detector distance of 239.6 mm. A silicon standard was used for calibration. The precision of the measurements was 3–5%.

Cyclic voltammetry (CV) measurements were performed in a glove box under a nitrogen atmosphere using a three-electrode cell with platinum (Pt) wires as working and counter electrodes and a non-aqueous Ag/Ag+ electrode (Ag in a 0.1 M AgNO_3_ solution) as the reference electrode in a solution of 0.1 M tetrabutylammonium hexafluorophosphate (TBAPF_6_) in anhydrous acetonitrile at typical scan rates of 20, 50, and 100 mV s^−1^. The cell was connected to a PA4 polarographic analyzer (Laboratory Instruments, Prague, Czech Republic) located outside the glove box.

### 2.4. Synthesis of Copolymers

#### 2.4.1. Poly{4,7-bis(4′-(2-octyldodecyl)thiophen-2′-yl)-5,6-difluorobenzo[*c*][1,2,5]thiadiazole-5′,5′-diyl-*alt*-9-(2-ethylhexyl)carbazole-2,7-diyl} (CP1)

The monomers 9-(2-ethylhexyl)-2,7-bis(4,4,5,5-tetramethyl-1,3,2-dioxaborolan-2-yl)carbazole (**1**, 0.30 mmol, 0.1594 g) and 4,7-bis(5′-bromo-4′-(2-octyldodecyl)thiophen-2′-yl)-5,6-difluorobenzo[*c*][1,2,5]thiadiazole (**3**, 0.30 mmol, 0.3166 g) were weighted into a glass reactor equipped with reflux condenser, magnetic stirrer, septum, and argon/vacuum inlet. Aqueous argon-degassed 15 wt.% NaHCO_3_ (10 mL), Aliquat 336 (20 mg), and argon-degassed xylene (7.0 mL) were added, and the reaction apparatus was degassed, repeating the vacuum/argon cycles (5×). Then the catalyst Pd(PPh_3_)_4_ (7.0 mg, 1 mol% per both monomers) in degassed xylene (3 mL) was added via septum, and the red reaction mixture was heated for 90 min at 131 °C under argon with efficient stirring.

Copolymer CP1-a: After cooling, the colorless water layer was separated and the organic layer was poured into methanol (700 mL). The red polymer was transferred into acetone (700 mL), stirred for 3 h, then filtered off (S2), and vacuum dried for 24 h. Yield: 0.3183 g (90%). The raw copolymer CP1-a was reprecipitated from toluene (30 mL) into acetone/methanol (600/300 mL); the red polymer was filtered off (S2) and dried to a constant weight (2 days). Yield: 0.2865 g (81%) of CP1-a. *M*_w_ = 78,600, *M*_n_ = 44,900.

Copolymers CP1-b and CP1-c: The reaction was end-capped first with phenylboronic acid pinacol ester (0.05 g, 0.25 mmol) for 15 min at 131 °C and second with 2-ethylhexyl bromide (0.20 mL, 1.12 mmol, ρ = 1.086 g/mL) for 15 min at 131 °C. After cooling, the colorless water layer was separated, and the organic layer was poured into methanol (800 mL). After sedimentation, the dark red polymer was filtered off (S2) and dried. Yield: 0.2983 g (85%). The polymer was reprecipitated from toluene (20 mL) into methanol (800 mL). Yield: 0.1265 g (36%) of CP1-b. *M*_w_ = 24,200, *M*_n_ = 17,200. To the filtrate, NaCl (5.0 g) was added, and the second portion of precipitate was filtered off (S2), thoroughly washed with water and methanol, and dried. Yield: 0.1552 g (44%) of CP1-c. *M*_w_ = 17,400, *M*_n_ = 10,500. The FTIR, ^1^H, and ^13^C NMR spectra of copolymers CP1-a, CP1-b, and CP1-c were found to be the same. Spectra for CP1-a are shown. FTIR (ATR): 2954, 2920, 2852, 1600, 1488, 1456, 1434, 1376, 1328, 1250, 1178, 1078, 1000, 894, 852, 804, 722, 654, 534 cm^−1^. ^1^H NMR (300.13 MHz, CDCl_3_, 295 K, δ): 8.20–7.10 (m, 8H, aromatic H), 4.21 (br s, 2H, Cz-N−CH_2_−), 2.82 (br s, 4H, 2 × Th−CH_2_−), 2.14 (br s, 2H, 2 × Th−C−CH<), 1.76 (br s, 3H, Cz-N−C−CH−CH_2_−Me), 1.60–1.00 (m, 70H, 35 × CH_2_), 1.00–0.70 (m, 18H, 6 × CH_3_); (found: sum of aromatic integrals Σ_arom_ = 8.33, sum of aliphatic integrals Σ_aliph_ = 106.18, Σ_aliph_/Σ_arom_ = 12.7; calcd. = 99H/8H = 12.4); (Figure S1). ^13^C NMR (75.45 MHz, CDCl_3_, δ): 151.7, 151.5, 149.1, 148.3, 148.0, 143.8, 141.5, 138.4, 134.2, 131.9, 129.6, 122.3, 121.2, 120.4, 111.5, 110.1 (all aromatic C), 47.7, 39.6, 39.2, 33.5, 33.4, 32.0, 31.2, 30.1, 29.8, 29.7, 29.4, 29.0, 26.6, 23.2, 22.8, 22.7, 14.2, 11.1 (all aliphatic C); (Figure S2).

#### 2.4.2. Poly{4,7-bis(4′-(2-octyldodecyl)thiophen-2′-yl)-5,6-difluorobenzo[*c*][1,2,5]thiadiazole-5′,5′-diyl-*alt*-dibenzothiophene-5,5-dioxide-3,7-diyl} (CP2)

The monomers 3,7-bis(4,4,5,5-tetramethyl-1,3,2-dioxaborolane-2-yl)dibenzothiophene-5,5-dioxide (**2**, 0.30 mmol, 0.1405 g) and 4,7-bis(5′-bromo-4′-(2-octyldodecyl)thiophen-2′-yl)-5,6-difluorobenzo[*c*][1,2,5]thiadiazole (**3**, 0.30 mmol, 0.3166 g) were weighted into a glass reactor equipped with reflux condenser, magnetic stirrer, septum, and argon/vacuum inlet. Aqueous argon-degassed 15 wt.% NaHCO_3_ (10 mL), Aliquat 336 (20 mg), and argon-degassed xylene (7.0 mL) were added, and the reaction apparatus was degassed, repeating the vacuum/argon cycles (5×). Then the catalyst Pd(PPh_3_)_4_ (7.0 mg, 1 mol% per both monomers) in degassed xylene (3 mL) was added via septum, and the red reaction mixture was heated for 90 min at 131 °C under argon with efficient stirring. The reaction was end-capped with: (a) phenylboronic acid pinacol ester (0.05 g, 0.25 mmol) for 15 min at 131 °C; (b) 2-ethylhexyl bromide (0.20 mL, 1.12 mmol, ρ = 1.086 g/mL) for 15 min at 131 °C. After cooling, the colorless water layer was separated, and the organic dark red layer was poured into acetone/MeOH (700/200 mL) to form a dark red precipitate, which was filtered off (S3) and dried (24 h). Yield: 0.3160 g of raw CP2 (95%). This material was reprecipitated from toluene (15 mL) into acetone/MeOH (500/400 mL). The dark red precipitate was filtered off (S3) and dried. Yield: 0.1932 g (58%) of CP2. *M*_w_ = 17,800, *M*_n_ = 8900. FTIR (ATR): 2952, 2920, 2852, 1592, 1532, 1466, 1438, 1314, 1158, 1078, 1010, 894, 854, 826, 782, 748, 716, 680, 620, 582, 534 cm^−1^. ^1^H NMR (300.13 MHz, CDCl_3_, 295 K, δ): 8.25–7.18 (m, 8H, aromatic H), 2.72 (br s, 4H, 2 × Th−CH_2_−), 1.72 (br s, 2H, 2 × Th−C−CH<), 1.23 (br s, 64H, 32 × CH_2_), 0.83 (s, 12H, 4 × CH_3_); (found: sum of aromatic integrals Σ_arom_ = 7.68, sum of aliphatic integrals Σ_aliph_ = 80.71, Σ_aliph_/Σ_arom_ = 10.5; calcd. = 82H/8H = 10.3); (Figure S3). ^13^C NMR (75.45 MHz, CDCl_3_, δ): 151.4, 148.1, 142.5, 139.7, 138.2, 136.9, 134.7, 131.0, 129.6, 128.6, 122.4, 121.8, 110.8 (all aromatic C), 39.3, 34.9, 33.5, 32.0, 30.1, 29.8, 29.4, 26.6, 22.8, 14.2 (all aliphatic C); (Figure S4).

## 3. Results and Discussion

### 3.1. Synthesis of Copolymers

**Scheme 1:**
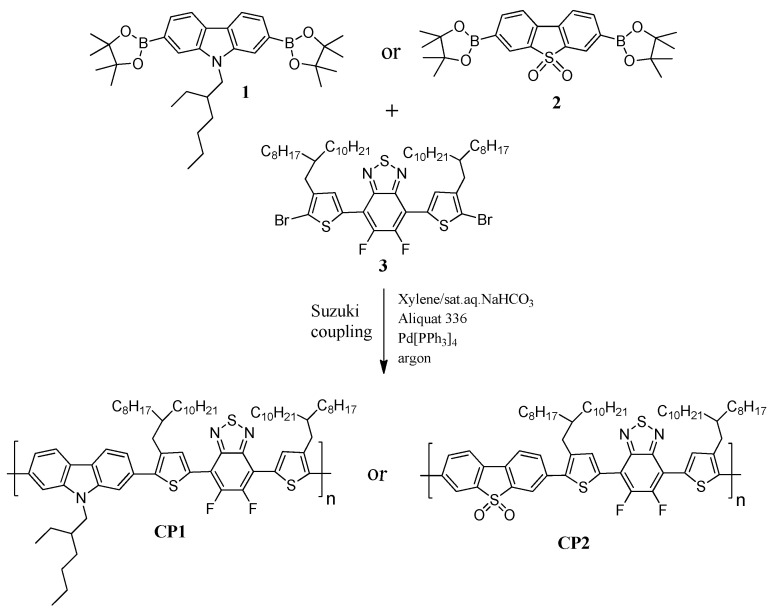
Synthesis of copolymers CP1 or CP2 by Suzuki coupling using monomers **1** or **2** and **3**.

The D-A copolymers CP1 or CP2 were synthesized by Suzuki coupling (xylene/sat. aq. NaHCO_3_, Pd(PPh_3_)_4_ as a catalyst, Aliquat 336 as a phase transfer agent, 131 °C, 90 min) of corresponding donor (**1** or **2**) and acceptor (**3**) comonomers (Scheme 1).

**Figure 2:**
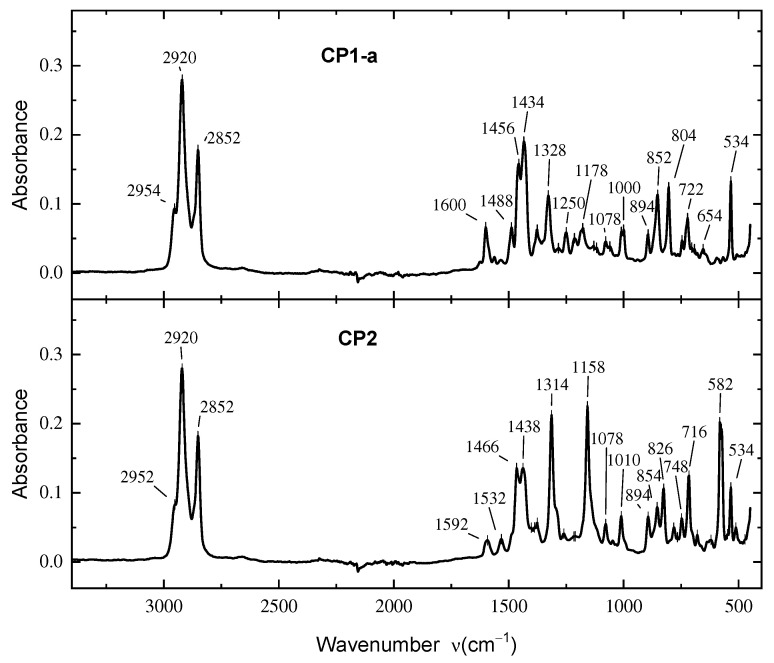
FTIR spectra of copolymers CP1-a and CP2.

The polymerization proceeded smoothly without (CP1-a) or with (CP1-b, CP1-c, and CP2) end-capping (phenylboronic acid pinacol ester, 2-ethylhexyl bromide), and the raw yields of all polymerizations were high (80–90%). The purification of polymers is described in more detail in the experimental part, and the reduced yields after the purification process are shown in Table 1. The synthesized D-A copolymers were characterized by FTIR (Figure 2), ^1^H and ^13^C NMR (Figures S1–S4) spectroscopies, and by size-exclusion chromatography (SEC, *M*_w_, *M*_n_, *Ð = M*_w_/*M*_n_, *P*) in THF (Table 1). Copolymers (CP1) with various molecular weights were obtained. The copolymer CP1-a with the highest weight-average molecular weight (*M*_w_ = 78,600) and a low dispersion was obtained without end-capping.

The FTIR spectra of copolymers CP1-a, CP1-b, and CP1-c were found to be identical. For instance, the FTIR spectrum of CP1-a (Figure 2) exhibited vibration bands at 2954, 2920 cm^−1^ (assigned to aromatic CH stretching), 2852 cm^−1^ (assigned to aliphatic CH stretching), 1600, 1488 cm^−1^ (assigned to aromatic ring stretching), 1456, 1434 cm^−1^ (assigned to the aliphatic CH_2_ scissors vibration and CH_3_ antisymmetric deformation), 1376 (CH_3_ symmetric deformation), 1328, 1250 cm^−1^ (assigned to carbazole N−C stretching), 1178, 1078, 1000 cm^−1^ (assigned to in-plane CH deformation), 894, 852, 804 cm^−1^ (assigned to out-of-plane CH deformation), 722 cm^−1^ (assigned to CH_2_ rocking in alkyls), 654 cm^−1^ (assigned to in-plane ring deformation), and 534 cm^−1^ (assigned to C−F stretching). The FTIR spectrum of copolymer CP2 (Figure 2) exhibited vibration bands at 2952, 2920 cm^−1^ (assigned to aromatic CH stretching), 2852 cm^−1^ (assigned to aliphatic CH stretching), 1592, 1532 cm^−1^ (assigned to aromatic ring stretching), 1466, 1438 cm^−1^ (assigned to the aliphatic CH_2_ scissors vibration and CH_3_ antisymmetric deformation), 1376 (CH_3_ symmetric deformation), 1314 cm^−1^ (SO_2_ antisymmetric stretching), 1158 cm^−1^ (SO_2_ symmetric stretching), 1078, 1010 cm^−1^ (assigned to in-plane CH deformation), 894, 854, 826 cm^−1^ (assigned to out-of-plane CH deformation), 748, 716 cm^−1^ (assigned to CH_2_ rocking in alkyls), 582 cm^−1^ (assigned to in-plane ring deformation), and 534 cm^−1^ (assigned to C−F stretching).

The ^1^H and ^13^C NMR spectra of copolymers CP1-a, CP1-b, and CP1-c were found to be nearly the same. In the ^1^H NMR spectrum of CP1-a (Figure S1), the peaks of the aromatic protons (8H) were found at 8.20–7.10 ppm, and the broad peaks of aliphatic protons were found at 4.21 (2H, Cz-N−CH_2_−), 2.82 ppm (4H, 2 × Th−CH_2_−), 2.14 (2H, 2 × Th−C−CH<), 1.76 (3H, Cz-N−C−CH−CH_2_−Me), and at 1.60–0.70 with more or less distinguished broad peaks of the CH_2_ and CH_3_ protons (88H). The measured ratio of the aliphatic to aromatic integrals, aliph.H/arom.H = 12.7 (theory: 99H/8H = 12.4), confirmed the structure of the repeating polymer unit of CP1, as shown in Scheme 1. In the ^13^C NMR spectrum of CP1-a (Figure S2), the peaks of aromatic (151.7–110.1 ppm) and aliphatic (47.7–11.1 ppm) carbons were clearly separated. In the ^1^H NMR spectrum of CP2 (Figure S3), the peaks of the aromatic protons (8H) were found at 8.25–7.18 ppm, and the broad peaks of aliphatic protons were found at 2.72 ppm (4H, 2 × Th−CH_2_−), 1.72 (2H, 2 × Th−C−CH<), 1.23 (64H, 32 × CH_2_), and at 0.83 (12H, 4 × CH_3_). The measured ratio of the aliphatic to aromatic integrals, aliph.H/aromH = 10.5 (theory: 82H/8H = 10.3), confirmed the structure of the repeating polymer unit of CP2, as shown in Scheme 1. In the ^13^C NMR spectrum of CP2 (Figure S4), the peaks of aromatic (151.4–110.9 ppm) and aliphatic (39.3–14.2 ppm) carbons were clearly separated.

### 3.2. Thermal Properties

**Figure 3:**
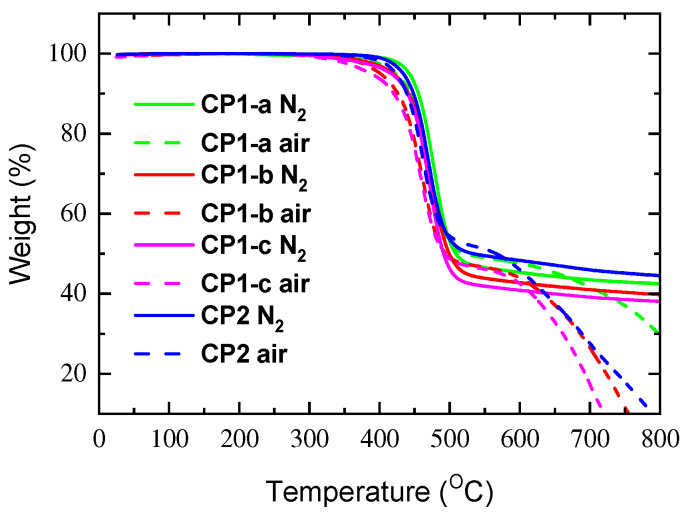
TGA curves of copolymers CP1-a, CP1-b, CP1-c, and CP2 measured at a scan rate of 10 K/min in N_2_ (solid) and in air (dashed).

TGA was used to investigate the thermal stability of the copolymers. Figure 3 shows TGA curves measured in N_2_ and in air at a heating rate of 10 K/min. From these curves, the temperatures at which 5% and 10% weight loss are observed and the residual weight percentages, which are given in Table 2, were evaluated.

Both CP1 and CP2 copolymers exhibited very good thermal and/or oxidation stability, as demonstrated by these weight loss results. The decrease is caused by additional decomposition of the alkyl chains on the thiophene rings and the alkyl chains on carbazole in the CP1 copolymers. The thermal stability of CP1 copolymers depended on their molecular weight. It decreased with its decline. The CP2 copolymer exhibited better thermal stability than the CP1-c copolymer with comparable molecular weight, possessing the 2-ethylhexyls in carbazole. The residual weight percentage reflecting the high aromatic content in N_2_ at 800 °C was 44% for CP2 and 38–42% for CP1, depending on the molecular weight; the higher value was for CP1-a with a higher molecular weight. The higher value for CP2 is in good agreement with the higher aromatic content in CP2. The copolymers showed very good thermal stability in the air as well.

### 3.3. Photophysical Properties

**Figure 4:**
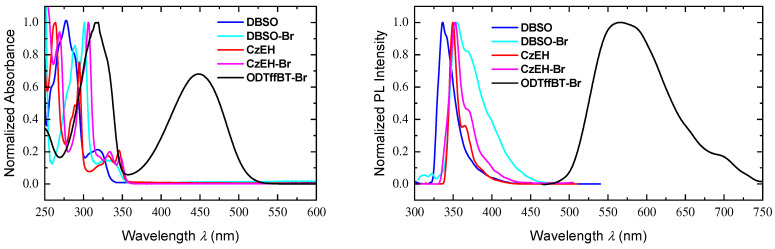
Normalized absorption (**left**) and photoluminescence (**right**) spectra of the CzEH and DBSO units, CzEH-Br, DBSO-Br, and ODTffBT-Br monomers measured in dilute THF solutions.

The photophysical properties of the copolymers were studied in dilute solutions and as thin films. In addition, the photophysical properties of the structural units CzEH, DBSO, and its brominated derivatives 9-(2-ethylhexyl)-2,7-dibromocarbazole (CzEH-Br), 3,7-dibromodibenzothiophene-5,5-dioxide (DBSO-Br), and monomer **3** (ODTffBT-Br) were also investigated for comparison and deeper understanding of photophysical processes in copolymers. Their absorption and PL spectra measured in THF solutions are displayed in Figure 4, and their maxima are summarized in Table 3.

CzEH and DBSO units absorbed UV light in spectral regions up to 355 and 335 nm, respectively. The CzEH spectrum shows typical spectral features for the 9-substituted carbazoles [72,73,74,75,76]. Bromination of the CzEH unit led to the red shift of the (0, 0) transition to the 2nd excited singlet state polarized along the long in-plane molecular axis of the carbazole unit (from 295 nm to 306 nm), whereas the maximum at 346 nm corresponding to (0, 0) transitions to the 1st excited singlet state polarized along the short molecular axis was nearly not influenced. The DBSO spectrum shows main bands with maxima at 279 and 290 nm and minor absorption at longer wavelengths with maxima at 314 and 322 nm, in accordance with literature [77]. Bromination of the DBSO unit also led to the red shift of absorption maxima, with less resolved minor absorption at longer wavelengths. Contrary to the absorption of CzEH-Br and DBSO-Br, the ODTffBT-Br absorption, consisting of structureless bands, is extended into the visible spectral region up to 520 nm. The lowest energy absorption band with a maximum at 448 nm is attributed to ICT between the central ffBT A unit and the 4-(2-octyldodecyl)thiophenes serving as D units in this ODTffBT structural block. The ICT transition maximum is significantly blue-shifted when compared with that at about 620 nm for the alkyl-substituted 4,6-di(thiophen-2′-yl)thieno [3,4-*c*][1,2,5]thiadiazole A units used in our previous work as building blocks in a series of D-A copolymers [38,39]. The PL spectra of the CzEH and DBSO units exhibit maxima at 349 and 337 nm, respectively. The PL emission maxima of CzEH-Br and DBSO-Br are red-shifted compared to those of the corresponding CzEH and DBSO units. The PL emission spectrum of ODTffBT-Br consists of a broad band in the visible spectral region with a maximum at 565 nm.

**Figure 5:**
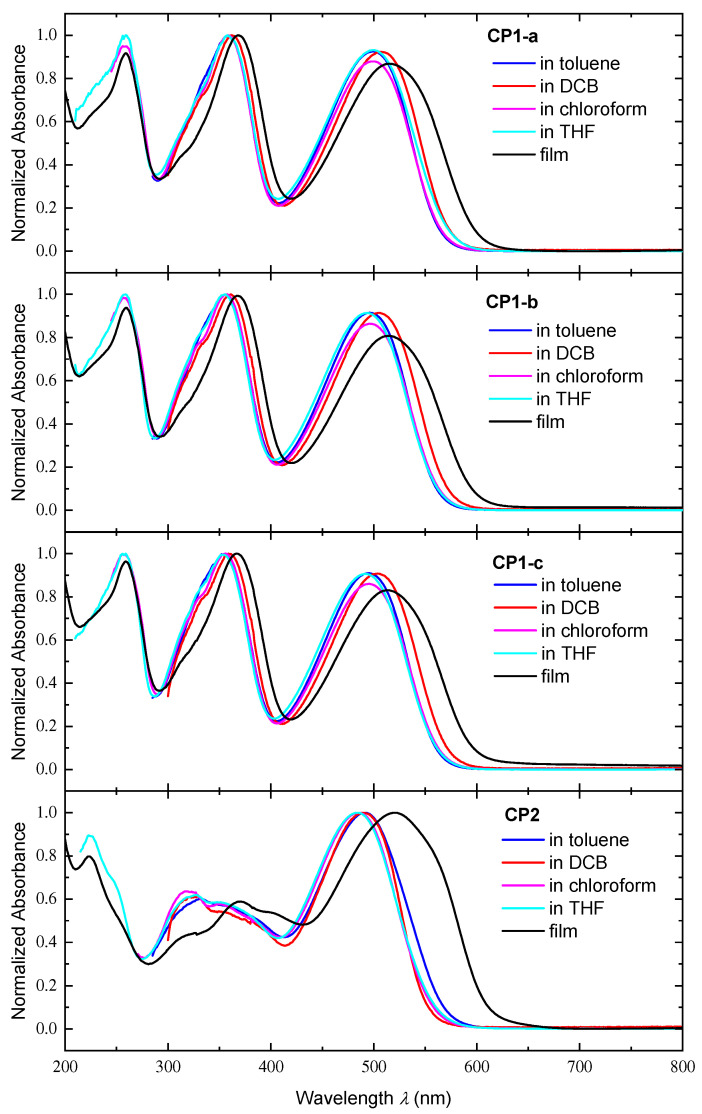
Normalized UV-vis absorption spectra of the CP1 and CP2 copolymers measured in dilute solutions and thin films.

**Figure 6:**
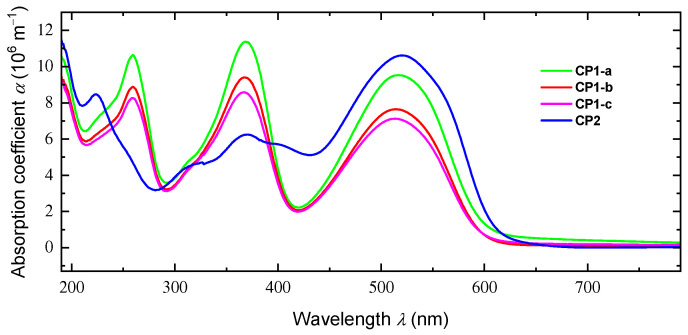
UV-vis absorption spectra of the CP1 and CP2 copolymers measured in thin films.

The absorption spectra of the copolymers measured in dilute solutions and in films are displayed in Figure 5 and Figure 6, and their maxima are summarized in Table 4 and Table 5. The CP1 spectra consist of three broad absorptions in the spectral regions of 200–300 nm, 300–400 nm, and 420–600 nm. The lowest-energy absorption band can be assigned to the ICT transition between the D unit and ffBT A moiety. Compared with the ODTffBT-Br monomer spectrum, a red shift of the ICT maximum, which is located at 495–499 nm in toluene, is observed due to the extended conjugation. The second lowest-energy absorption band with maxima at 354–359 nm in toluene can be assigned to the lowest π-π* transition of the conjugated backbone. The positions of both maxima slightly depended on the CP1 copolymer molecular weight. Blue shifts are observed in the spectra of CP1-b and CP1-c with the lower molecular weights compared with the highest-molecular-weight CP1-a. CP1 spectra measured in DCB are slightly redshifted compared with those measured in toluene, chloroform, and THF, which are similar to those measured in toluene solution. The absorption spectra of CP2 in solutions are similar for all solvents used, with only small differences in the positions of the maxima. The CP2 solution spectra are blue-shifted compared with those of CP1, which correlate well with the different electron-donating characters of the D unit. The ICT band in the CP2 spectra dominates, while in the CP1 spectra, the absorbance of the lowest π-π* transition maximum is higher than that of the ICT maximum.

The absorption spectra of thin films are red-shifted compared to the solution spectra. The red shifts of thin film absorption spectra compared to their solution spectra indicate an increased backbone planarity and/or formation of J-like aggregate in the solid state [78,79]. The most pronounced red shift observed for CP2 film spectra could indicate higher backbone planarity in the CP2 films compared to the CP1 films. The copolymers exhibited high values of the absorption coefficient *α* up to 10^7^ m^−1^ (see Figure 6). The *α* values of CP1-a films were higher than those of films made of lower-molecular-weight CP1-b and CP1-c copolymers, which could be due to differences in the supramolecular structure of the copolymer layers, as already mentioned above and discussed further in more details below. A similar influence of molecular weight on absorption coefficient was observed, for example, in films made of poly(9,9-dihexadecylfluorene-2,7-diyl-*alt*-2,2′-bithiophene-5,5′-diyl)s with different molecular weights [80]. CP2 layers showed the highest *α* values in the visible spectral region.

**Figure 7:**
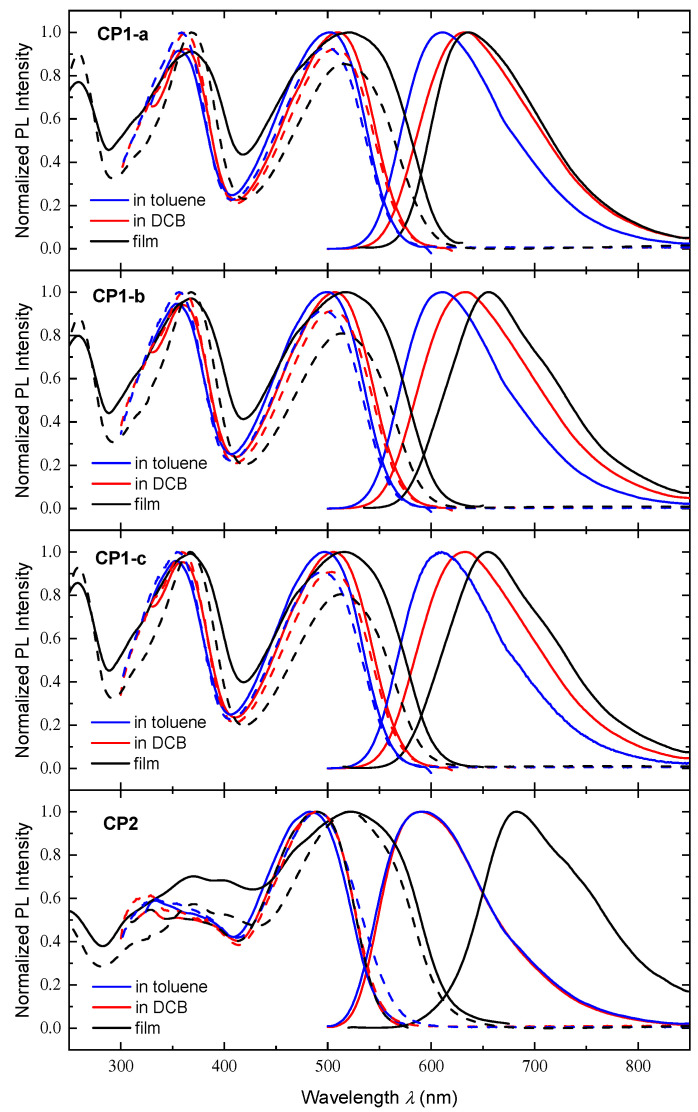
PL excitation and emission spectra of the copolymers under study measured in toluene and 1,2-dichlorobenzene dilute solutions and as thin films. Dashed lines display normalized absorption spectra for comparison with the excitation spectra.

Normalized PL spectra measured in dilute solutions and in thin films are displayed in Figure 7, and their maxima are summarized in Table 4 and Table 5. The CP1 spectra measured in toluene and DCB solutions are similar and nearly independent of molecular weight. The PL spectra measured in DCB exhibited maxima at 633–634 nm, which are red-shifted compared with those at 610–611 nm measured in toluene. The CP2 solution spectra are similar in both toluene and DCB solutions, with maxima at about 590 nm and being blue-shifted compared to the CP1 spectra. The blue shift reflects the weaker electron-donating character of the DBSO D unit in CP2 compared to the CzEH D unit in CP1 and is in accordance with the results of the absorption study. The shapes of the CP1 excitation spectra show similar features as the absorption spectra. A small difference in the intensity of the maxima corresponding to π-π* and ICT transitions was observed. In the PL excitation spectra, the intensity of both maxima is similar, whereas in the absorption spectra, the absorbance of the ICT maximum is lower than that of the corresponding π-π* transition. The shapes of the CP2 excitation spectra follow well the absorption ones.

In thin films, the PL emission spectra of all copolymers are red-shifted compared to those measured in dilute solutions. The PL spectra of CP1-a films with a maximum at 638 nm exhibited the smallest red shifts. The PL spectra of the films made of lower-molecular-weight CP1-b and CP1-c copolymers exhibited larger red shifts, with maxima at 654 nm indicating improved molecular planarity and more pronounced excimer or aggregate formation. The largest red shift was observed for CP2 film spectra, with the maximum at 682 nm, which correlates well with the results of the absorption study, where the largest red shift of the absorption bands was observed in the absorption spectra of CP2 thin films compared to their solution spectra.

High values of PL quantum yield (*η*_PL_) that were in the range 0.8–0.9, were evaluated for CP1 toluene and DCB solutions with no significant dependence on molecular weight. Lower *η*_PL_ values of 0.5 and 0.7 were found for CP2 toluene and DCB solutions, respectively. We introduced the relative PL efficiency of a thin film, *Φ*_Plrel_, to compare the PL efficiencies of the films. The highest *Φ*_Plrel_ values were evaluated for films made of low-molecular CP1-b and CP1-c. They were nearly two times higher than that for CP1-a, which has the highest molecular weight. The lowest *Φ*_Plrel_ values were found for CP2 films, which were lower by factor 3.5 than those for CP1-b and CP1-c. PL emission is efficiently quenched in thin films made of blends of the copolymers with electron acceptors such as [6,6]phenyl-C_71_-butyric acid methyl ester (PC_71_BM).

**Figure 8:**
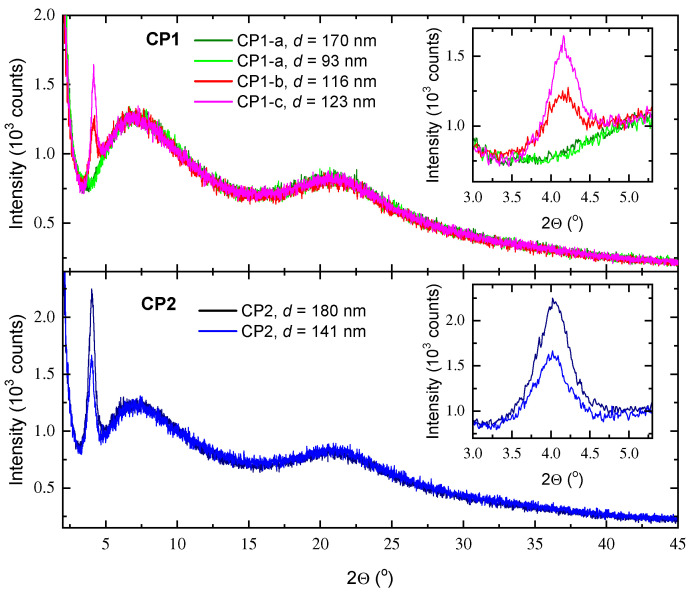
XRD patterns of thin copolymer films on fused silica substrates (*d* = film thickness). Insets show the XRD peaks in detail.

The observed differences in absorption and PL properties of the copolymers could be explained by differences in the polymer backbone planarity and/or molecular arrangement in the solid state, which are influenced not only by the copolymer chemical structure but also by the molecular weight in the case of the CP1 copolymers, as already mentioned above. Therefore, we have performed preliminary XRD measurements on thin films to find if there are any differences in the supramolecular structure. XRD patterns of thin copolymer films on fused silica substrates are displayed in Figure 8.

In the XRD patterns of the layers made of CP1-b and CP1-c, a sharp peak located at the angle 2θ = 4.16° was found, whereas any peak was not observed in the patterns of CP1-a layers, i.e., made of copolymer with the highest molecular weight (*M*_w_ = 78,600, see Table 1). Similarly, XRD patterns of CP2 layers exhibited a sharp peak at 2θ = 4.03°. The molecular weight of CP2 was *M*_w_ = 17,800, which is comparable to that of CP1-c (*M*_w_ = 17,400). The diffusive halos in all diffractograms corresponded to XRD from the fused silica substrate. The peak positions are in the range typical for lamellar stacking, i.e., scattering due to the lateral molecular separation between the polymer backbones. The peaks could be associated with the lamellar distance in the so-called “edge-on” orientation of the copolymer molecules, where the copolymer backbone axis is in the substrate plane and the backbone plane and alkyl chains are oriented normal to the substrate surface [81,82,83,84]. The lamellar distance *d* was evaluated using the Bragg relation n λ=2 dsin⁡θ, where *n* is the order of the interference, λ is the X-ray wavelength, and θ is the angle of incidence. A lamellar distance value of 2.12 nm was determined for both CP1-b and CP1-c, and a slightly larger *d* value of 2.19 nm was found for CP2 films. The larger *d* value in CP2 films could indicate the higher backbone planarity in CP2 films compared to CP1 films, which correlates well with the results of the photophysical study.

Further, we have evaluated the mean lamellar domain size from the line broadening, which is related to the crystallite (lamellar) domain size [85,86]. The larger domains enhance the peak intensity. The average domain size, *D*, was evaluated using the Scherrer equation as follows:

(1) $$D=\frac{K\lambda }{\beta \cos\theta }$$

where λ is the X-ray wavelength, β is the full width at the half maximum of the reflection peak located at angle 2θ after subtraction of the instrumental broadening ($\beta =\sqrt{\beta_{measured}^{2}-\beta_{intrumental}^{2}}$), and *K* is the Scherrer constant (a dimensionless factor depending on the crystallite shape and size distribution), which was found to be in the range of 0.62–2.08 [87]. If the crystallite shape and distribution are unknown, there is uncertainty in the *K* value. We have made our calculation with the typically used *K* value of 0.9. The largest lamellar domain size of 18 nm was determined for CP1-c, 14.8 nm for CP1-b, and 15 nm for CP2 layers.

### 3.4. Electrochemical Properties

**Figure 9:**
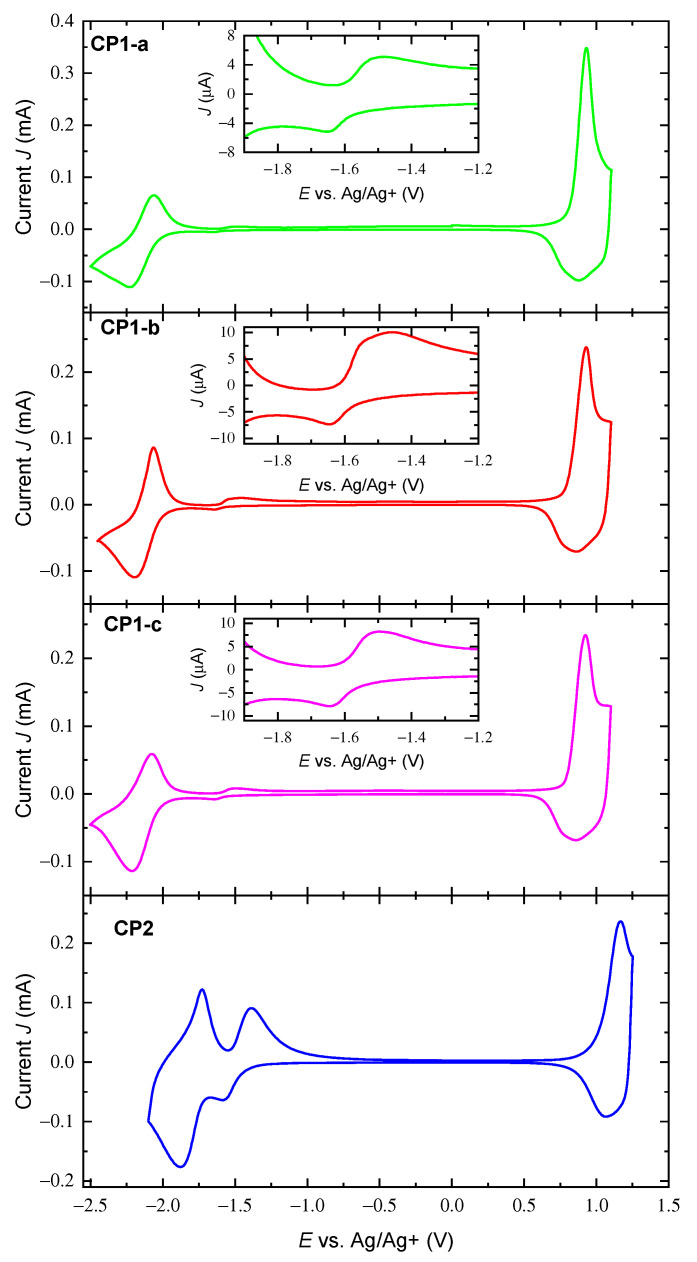
Representative cyclic voltammograms of copolymer thin films coated on a Pt wire recorded at a scan rate of 50 mVs^−1^. The first reduction peaks of CP1 copolymers are displayed in the insets.

CV was used to determine the electronic structure of the copolymers. Figure 9 displays representative CV curves of thin copolymer films on a Pt wire. The copolymers exhibited reversible oxidation and reduction. The peak separation Δ*E*_p_ was lower than 100 and 180 mV for oxidation and reduction processes, respectively, at a rate of 50 mV s^−1^, and it decreased with lower scan rates. In the CV curve of the CP2, two pairs of peaks at different potentials occurred in the reduction cycle, which indicated the presence of two redox reactions. The peak current of the first reduction peak is smaller than that of the second reduction peak. Two-wave one-electron reductions were not clearly observed in the CV curves of CP1 copolymers, where the first reduction peaks were significantly suppressed. 

The ionization potential *E*_IP_ (-HOMO level) was estimated from the onset of oxidation potential and the electron affinity *E*_A_ (-LUMO level) from the onset of the first reduction peak using the equation *E*_IP_ (*E*_A_) = |−(*E*_onset_ – *E*_ferr_) – 4.8| eV, where *E*_onset_ is the corresponding onset potential and *E*_ferr_ is the half-wave potential for ferrocene (vs. Ag/Ag^+^), which was used as a standard with a reference energy level of 4.8 eV below the vacuum level. The *E*_IP_ and *E*_A_ values were calculated from several CV curves measured at a scan rate of 50 mVs^−1^, and their average values are given in Table 6. The CP2 copolymer exhibited higher ionization potential (*E*_IP_ = 5.72 eV) and also higher values of electron affinity than those of the CP1 copolymers (*E*_IP_ = 5.54–5.55 eV), which correlates well with the stronger electron-donating capability of the CzEH unit than that of the DBSO unit. The electrochemical bandgap values, *E*_g_^elc^, were similar for all copolymers of approximately 2.3–2.4 eV, which are higher values than the optical bandgap values determined for direct allowed transition from the absorption spectra of thin films (^f^*E*_g_^opt-d^) and also slightly higher than those evaluated from the solution absorption spectra in toluene (^tol^*E*_g_^opt-d^). These optical bandgap values for direct transition were evaluated from the dependence of the absorption coefficient α on energy E according to the relation $\alpha =\alpha_{0}{\left(h\upsilon -E_{g}\right)}^{1/2}$, where *hυ* is photon energy and *E*_g_ is the energy bandgap (the extrapolation of the linear dependence of $\alpha^{2}\;vs.\;E$ to α = 0).

### 3.5. Electroluminescence

**Figure 10:**
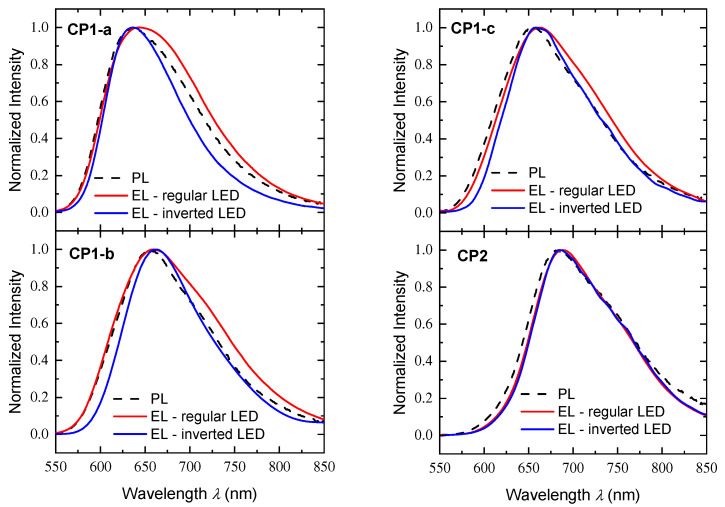
Normalized EL spectra of the regular ITO/PEDOT:PSS/copolymer/Ca/Al (red) and inverted ITO/ZnO/copolymer/MoO_3_/Ag (blue) LEDs made of CP1 and CP2 copolymer active layers. The PL spectra of thin films are displayed as dashed lines for comparison.

The copolymers were tested as active layers in LEDs with regular and inverted structures. The LEDs of regular structure (regular LEDs) consisted of ITO covered with a PEDOT:PSS layer as a hole-injecting electrode and Ca covered with Al (Ca/Al) as an electron-injecting electrode, whereas the LEDs with inverted structure (inverted LEDs) were composed of ITO covered with ZnO and MoO_3_ layers covered with Ag layers, respectively, forming the electron- and hole-injecting electrodes. The EL spectra of the LEDs are shown in Figure 10. All LEDs exhibited an intense red EL emission. The EL spectra show similar shapes when compared with the PL spectra of thin films. For the LEDs made of CP1 copolymers, the EL spectra of inverted LEDs slightly differ compared with those of regular LEDs. In the case of CP2 LEDs, nearly no differences in the EL spectra shapes for both structures were observed. No significant differences in the PL and EL spectra indicate that PL and EL processes have similar origins. The maxima of the EL spectra of regular and inverted LEDs were located at 643 and 637 nm for CP1-a, at 658 and 660 nm for CP1-b, 660 and 658 nm for CP1-c, and at 688 and 685 nm for CP2, respectively. Some differences in the shapes of the EL spectra appeared for the different voltages. Usually, broader EL spectra with a slight red shift in maxima are observed at lower voltages. The differences in the EL spectra of the regular and inverted LEDs could be explained in part by the resonant effect due to the different optical parameters of the device layers [88,89,90]. Spectral shapes and maximum positions could also be influenced by differences in the active layer morphology. The EL spectra of CP1-a LEDs differ from those of LEDs made of CP1-b and CP1-c, similarly to the PL spectra. The differences in the morphology of layers prepared from CP1-a with the highest molecular weight when compared with CP1-b, CP1-c, and CP2 were found as already discussed above.

**Figure 11:**
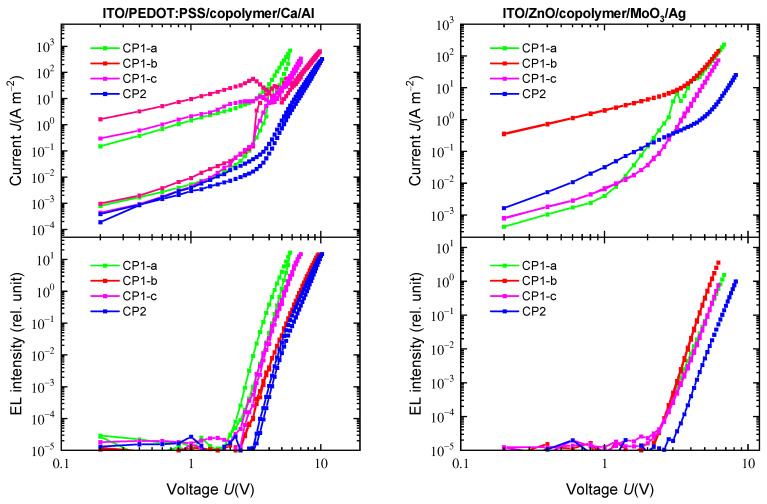
Dependence of current density and EL intensity on applied voltage of the regular ITO/PEDOT:PSS/copolymer/Ca/Al (**left**) and inverted ITO/ZnO/copolymer/MoO_3_/Ag (**right**) LEDs made of the CP1 and CP2 copolymer active layers.

The regular and inverted LEDs exhibited good performance with low onset voltages between 2 and 3 V. Examples of the current-voltage and EL intensity-voltage characteristics of LEDs are displayed in Figure 11. At low applied voltages *U*, the current *j* increased linearly or with a slightly higher exponent up to 2. At the region of the onset voltage, when EL emission begins, the current starts to increase steeper. At higher voltages, the current increases sharply as *j~U^m^* with an *m* of 6–10. Compared to the current–voltage dependence, the EL intensity (*I*_EL_)–voltage dependence is even steeper. *I*_EL_ increases as *I*_EL_*~U^n^*, with an *n* > 10. Anomalous behavior was observed in the current-voltage characteristics of LEDs; therefore, the current at low voltages (<the onset voltage) could have different values that depended on the history of the applied voltage. The anomalous behavior was observed in LEDs made of conjugated polymers, and a reversible memory effect, including the existence of two states with low and high resistance at low voltages, whose appearance depended on the history of the applied voltage, was revealed [88,91]. Switching between the low and high resistance states can be realized by applying a voltage pulse or switching off the applied voltage in a certain voltage region, as described in our previous papers. For example, in our case here, when a voltage of approximately >4 V is applied and switched off, a low current will subsequently be measured at low voltages (high resistance state) up to a voltage with a sharp increase in current, when a high current will be subsequently measured at low voltages (low resistance state). This low resistance state remains even when the applied voltage is turned off (at low voltages) until it is reset again, i.e., switched to the high resistance state by turning off the higher applied voltage as described above. Materials that exhibit anomalous behavior with memory effects are interesting for bio-inspired electronics as an example of artificial synapsis that could detect chemicals or respond to light, an electric field, or other physical stimuli [92,93,94]. In the LEDs with CP1 active layers, the relative EL efficiency was higher for the regular LEDs than that for the inverted LEDs. The CP2 inverted LEDs exhibited higher EL efficiency at low current densities than the CP2 regular LEDs. LEDs of the simple single-layer structure without any optimization were tested only, and therefore their efficiency could be further enhanced, for example, by the optimization of the active layer using polymer blends and/or the LED architecture optimization [91,95,96,97,98,99,100].

### 3.6. Outlook for Photovoltaics

**Figure 12:**
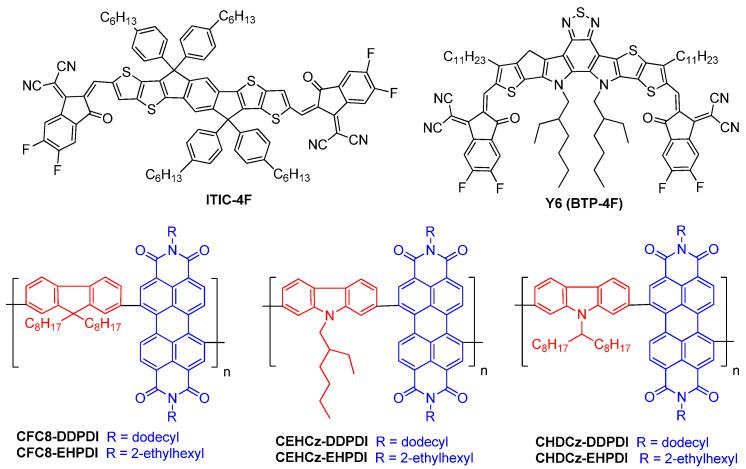
Chemical structure of two small molecule electron acceptors, ITIC-4F and Y6, and of the n-type D-A copolymers poly[*N*,*N*′-dialkylperylene-3,4,9,10-tetracarboxydiimide-1,7-diyl-*alt*-9,9-dioctylfluorene-2,7-diyl or 9-alkylcarbazole-2,7-diyl]s.

The results of the studies showed the potential of the CP1 and CP2 copolymers for photovoltaic applications, especially for organic or perovskite solar cells. In organic solar cells, they could be used mainly as electron donors in blends with one or two electron acceptors in binary or ternary solar cells. The acceptors could be either fullerene derivatives, non-fullerene organic electron acceptor molecules, or n-type polymers with suitable properties to form bulk heterojunctions. Non-fullerene small-molecule organic electron acceptors such as ITIC-4F or Y6 (Figure 12) can be used. D-A copolymers with perylene-3,4,9,10-tetracarboxydiimide-based A units and various D units (Figure 12) synthesized and studied in our previous work are suitable as electron acceptors due to their higher electron affinities (3.8–3.9 eV) and other desirable properties [46,47,101]. These n-type polymers could also be used in ternary solar cells together with a second acceptor, such as ITIC-4F or Y6, with higher electron affinities and absorption in the NIR region, improving sunlight harvesting. Additionally, the energy transfer from the donor copolymers to the acceptors may also occur as an important competitive process for electron transfer [102]. The electron acceptor Y6 may be even more interesting than ITIC-4F [103]. The results of the EL study performed on both regular and inverted LEDs indicated that both electrode structures are suitable for utilization in solar cells as collecting electrodes. The performance and stability of inverted samples could be further improved by modification of the ZnO layer using 2-(3-(dimethylamino)propyl)-1,3-dioxo-2,3-dihydro-1*H*-benzo[*de*]isoquinoline-6,7-dicarboxylic acid [104]. In addition, the CP1 and CP2 copolymers could also be useful for perovskite solar cells, as already mentioned in the introduction.

## 4. Conclusions

Two new copolymers, CP1 and CP2, possess the same 4,7-bis(4′-(2-octyldodecyl)thiophen-2′-yl)-5,6-difluorobenzo[*c*][1,2,5]thiadiazole blocks with the central ffBT A unit and different D units, CzEH in CP1 with stronger electron-donating ability than DBSO in CP2, were synthesized by Suzuki coupling in high-boiling solvent xylene and their properties investigated. Both CP1 and CP2 copolymers exhibited very good thermal and oxidation stability. Higher values of ionization potential and electron affinity were found for CP2 than for CP1, which correlate well with the electron-donating ability of corresponding D units. A CP1 copolymer with different molecular weights (CP1-a, CP1-b, and CP1-c) was prepared, which allowed us to reveal the effects of molecular weight on the CP1 properties and the direct comparison of both copolymers (CP1-c and CP2) of similar molecular weights. The CP1 copolymers exhibited efficient red PL emission in dilute toluene and DCB solutions with high PL quantum yields of 80–90% without any significant dependence on molecular weight. A more pronounced influence of molecular weight was found for thin films, in which aggregate formation is evident. Films made from the lower-molecular-weight CP1-b and CP1-c showed higher PL efficiency than films made of the highest-molecular-weight CP1-a. The PL spectra of CP1-a films were blue-shifted compared to those of CP1-b and CP1-c films, indicating differences in aggregate formation. The copolymer CP2 showed lower PL quantum yield in its solutions as well as lower PL efficiency in thin films than CP1 copolymers. Compared to CP1, the PL spectra of CP2 solutions were blue-shifted, and conversely, the PL spectra of CP2 films were red-shifted, indicating better backbone planarity. The results of XRD measurements, which correlate well with the results of the photophysical study, showed that molecular weight influences molecular arrangement in thin films. Significant differences were found in the supramolecular structure of films made from the highest-molecular-weight CP1-a and the films made from the lower-molecular-weight CP1-b, CP1-c, and CP2 copolymers, where a lamellar structure with domain sizes of about 15–18 nm was observed. A longer lamellar distance of 2.19 nm was found for CP2 than the distance of 2.12 nm for both CP1-b and CP1-c. LEDs with active layers made of both new copolymers exhibited efficient red emission with low onset voltages. Anomalous current–voltage behavior with memory effects observed in their characteristics could also be of interest for future photonic and bio-inspired electronic applications. Efficient PL quenching observed in the thin films made of blends with the electron acceptor also indicated the potential of new copolymers for photodetector and photovoltaic applications. The detailed study provided direct insight into the photophysics, electronic structure, and electroluminescence of new copolymers and also indicated their potential for possible applications in photonics and electronics.

## Figures and Tables

**Table 1:** Size-exclusion chromatography (SEC) data ^a^ (*M*_w_ is the weight-average molecular weight, *M*_n_ the number-average molecular weight, *Đ* the dispersity, *P* the polymerization degree) and yields (after purification) for the D–A copolymers CP1-a, CP1-b, CP1-c, and CP2.

Copolymer	Yield (%)	End Capping	*M*w	*M*n	*Ð*	*P*
CP1-a	81	−	78,600	44,900	1.75	67
CP1-b	36	+	24,200	17,200	1.41	21
CP1-c	44	+	17,400	10,500	1.66	15
CP2	58	+	17,800	8900	2.00	16

^a^ Measured by SEC in tetrahydrofuran as the mobile phase, polystyrene standards were used for calibration.

**Table 2:** Thermal properties of copolymers (*T*_d5%_ and *T*_d10%_ are the decomposition temperatures at which 5% and 10% weight loss were recorded, respectively, and *W*_res_ is the residual weight percentage at 800 °C under N_2_).

Copolymer	*M*_w_	*Đ*	*T*_d5%_ (°C)		*T*_d10%_ (°C)		*W*_res_ (%)
			N_2_	Air	N_2_	Air	N_2_
CP1-a	78,600	1.75	442	422	455	443	42
CP1-b	24,200	1.41	419	401	441	426	39
CP1-c	17,400	1.66	416	397	440	420	38
CP2	17,800	2.00	433	423	448	439	44

**Table 3:** Photophysical properties of the structural units and monomers measured in diluted THF solutions (*λ*_absmax_ absorption maximum, *λ*_PLmax_ PL emission maximum, *λ*_Plexcmax_ maximum of excitation spectrum). The main maxima are printed in bold.

Structural and Comonomer Units	*λ*_absmax_ (nm)	*λ*_Plmax_ ^a^ (nm)	*λ*_Plexcmax_ ^b^ (nm)
EHCz	**264**, 295, 331, 346	349	**261**, 294, 331, 344
EHCz-Br	**269**, 306, 334, 347	352	266, **304**, 333, 348
DBSO	**279**, 290, 314, 322	337	**278**, 289, 321
DBSO-Br	289, **301**, 331	355	287, **300**, 339
ODTffBT-Br	249, **319**, 448	565	**316**, 448

^a^ Excitation wavelength at *λ*_absmax_; ^b^ Emission wavelength at *λ*_Plmax._

**Table 4:** Photophysical properties of copolymers under study measured in dilute solutions (*λ*_absmax_, absorption maximum; *λ*_Plmax_, PL emission maximum; *λ*_Plexcmax_, maximum of excitation spectra; *η*_PL_, PL quantum yield). The main maxima are printed in bold.

Copolymer	Solvent	*λ*_absmax_ (nm)	*λ*_Plmax_ ^a^ (nm)	*λ*_Plexcmax_ ^b^ (nm)	*η*_PL_
CP1-a	toluene	**359**, 499	611	356, **502**	0.83 ± 0.07
CP1-b	toluene	**356**, 495	611	354, **499**	0.93 ± 0.07
CP1-c	toluene	**354**, 495	610	353, **497**	0.90 ± 0.07
CP2	toluene	331, **490**	592	333, **482**	0.52 ± 0.04
CP1-a	DCB	**363**, 507	634	363, **510**	0.91 ± 0.07
CP1-b	DCB	**361**, 505	633	361, **507**	0.86 ± 0.07
CP1-c	DCB	**359**, 504	633	359, **506**	0.89 ± 0.07
CP2	DCB	319, **488**	590	330, **489**	0.70 ± 0.06

^a^ Excitation wavelength was 485 nm, ^b^ Emission wavelength was *λ*_Plmax_.

**Table 5:** Photophysical properties of the polymers measured in thin films (*λ*_absmax_, absorption maximum; *λ*_Plmax_, PL emission maximum; *λ*_Plexcmax_, maximum of excitation spectra; *Φ*_Plrel_, relative PL efficiency). The main maxima are printed in bold.

Copolymer	*λ*_absmax_ (nm)	*λ*_Plmax_ ^a^ (nm)	*λ*_Plexcmax_ ^b^ (nm)	*Φ*_Plrel_
CP1-a	259, **368**, 516	638	259, 368, **521**	0.52 ± 0.04
CP1-b	259, **368**, 514	654	259, 367, **518**	0.93 ± 0.07
CP1-c	259, **367**, 512	654	258, 367, **516**	1.00
CP2	224, 366, **520**	682	248, 370, **522**	0.28 ± 0.02

^a^ Excitation wavelength at *λ*_absmax_; ^b^ Emission wavelength at *λ*_Plmax._

**Table 6:** Electronic properties of the copolymers (*E*_IP_: ionization potential; *E*_A_: electron affinity; *E*_g_^elc^: electrochemical bandgap; ^f^*E*_g_^opt-d^ and ^tol^*E*_g_^opt^: optical bandgap evaluated from absorption spectra of films and toluene solution, respectively).

Copolymer	*E*_IP_ (eV) (−*E*_HOMO_)	*E*_A_ (eV) (−*E*_LUMO_)	*E*_g_^elc^ (eV)	^f^*E*_g_^opt-d^ (eV)	^tol^*E*_g_^opt-d^ (eV)
CP1-a	5.55	3.22	2.33	2.12	2.26
CP1-b	5.54	3.21	2.33	2.15	2.27
CP1-c	5.54	3.21	2.33	2.15	2.27
CP2	5.72	3.33	2.39	2.07	2.27

## Data Availability

Data are contained within the article and supplementary materials.

## Supplementary Materials

The following supporting information can be downloaded at: https://www.mdpi.com/article/10.3390/nano13222939/s1. Figure S1: ^1^H NMR spectrum of copolymer CP1-a; Figure S2: ^13^C NMR spectrum of copolymer CP1-a; Figure S3: ^1^H NMR spectrum of copolymer CP2; Figure S4: ^13^C NMR spectrum of copolymer CP2.
