# Supplementary material for: Donor-Acceptor Copolymers with 9-(2-Ethylhexyl)carbazole or Dibenzothiophene-5,5-dioxide Donor Units and 5,6-Difluorobenzo[c][1,2,5]thiadiazole Acceptor Units for Photonics
Source: Nanomaterials (Basel). 2023 Nov 13;13(22):2939. doi: 10.3390/nano13222939 (PMC10675554; doi:10.3390/nano13222939)
Supplement: Supplementary file 1 [file nanomaterials-13-02939-s001.zip › nanomaterials-2639141-supplementary.pdf]

Supplementary Materials

Donor-Acceptor Copolymers with 9-(2-Ethylhexyl)carbazole or Dibenzothiophene-5,5-dioxide Donor Units and 5,6-Difluorobenzo[*c*][1,2,5]thiadiazole Acceptor Units for Photonics

Věra Cimrová*, Petra Babičová, Mariem Guesmi and Drahomír Výprachtický

Institute of Macromolecular Chemistry, Czech Academy of Sciences, Heyrovského nám. 2, 162 00 Prague 6, Czech Republic

*Correspondence: cimrova@imc.cas.cz

Content

Page

Figure S1: ^{1}H NMR spectrum of copolymer CP1-a (spectra of CP1-a, CP1-b, and CP1-c were found to be similar). 3
Figure S2: ^{13}C NMR spectrum of copolymer CP1-a (spectra of CP1-a, CP1-b, and CP1-c were found to be similar). 3
Figure S3: ^{1}H NMR spectrum of copolymer CP2. 4
Figure S4: ^{13}C NMR spectrum of copolymer CP2. 4

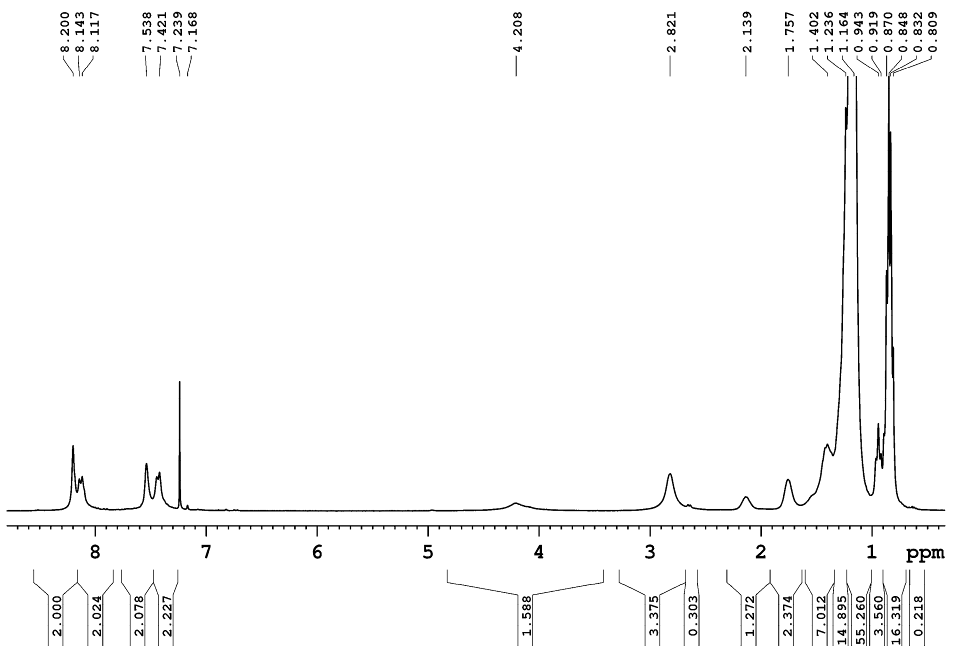

Figure S1. ^{1}H NMR spectrum of copolymer CP1-a (spectra of CP1-a, CP1-b, and CP1-c were found to be similar).

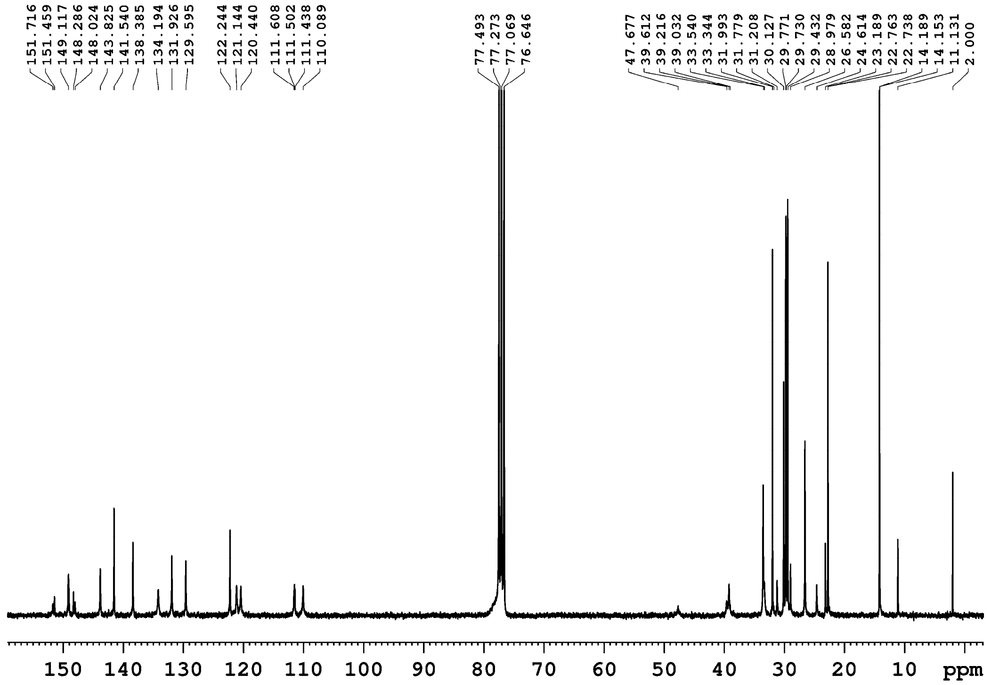

Figure S2. ^{13}C NMR spectrum of copolymer CP1-a (spectra of CP1-a, CP1-b, and CP1-c were found to be similar).

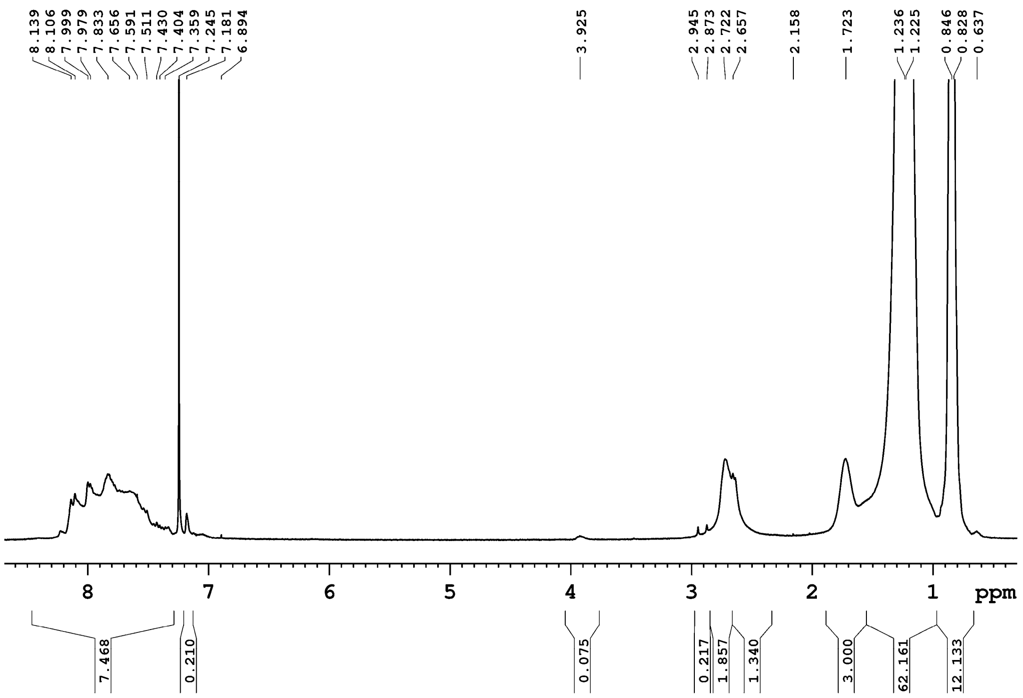

Figure S3. ^{1}H NMR spectrum of copolymer CP2.

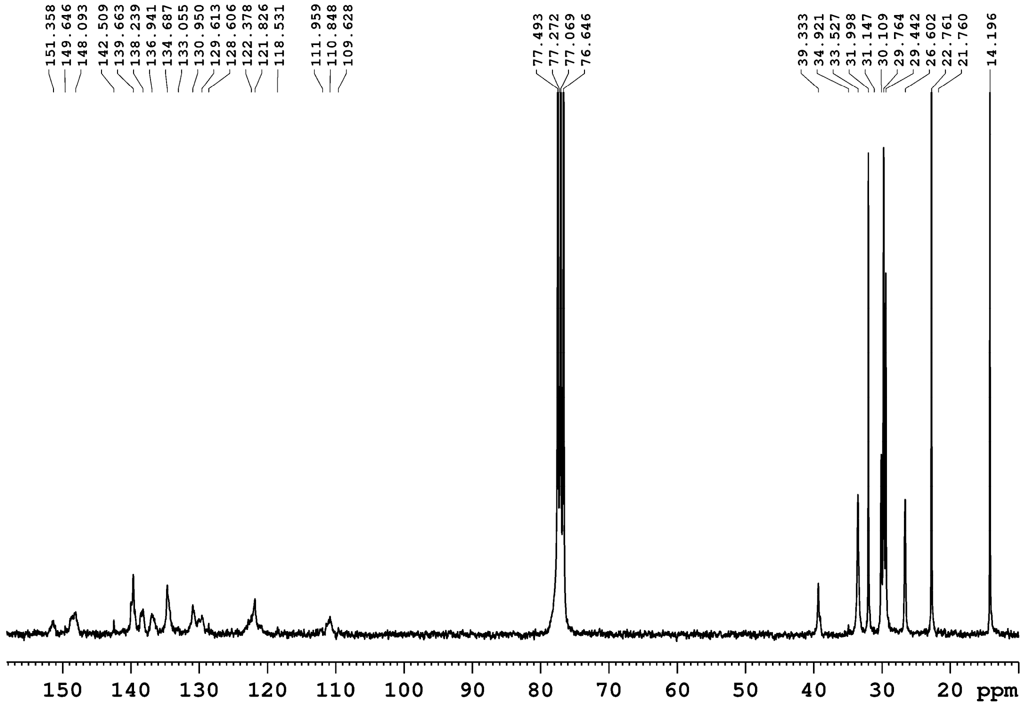

Figure S4. ^{13}C NMR spectrum of copolymer CP2.
